# Evaluation of an artificial intelligence–based algorithm for automated localization of craniofacial landmarks

**DOI:** 10.1007/s00784-023-04978-4

**Published:** 2023-04-04

**Authors:** Friederike Maria Sophie Blum, Stephan Christian Möhlhenrich, Stefan Raith, Tobias Pankert, Florian Peters, Michael Wolf, Frank Hölzle, Ali Modabber

**Affiliations:** 1 Department of Orthodontics, University Hospital of RWTH Aachen, Pauwelsstraße 30, D-52074 Aachen, Germany; 2 Department of Orthodontics, Witten/Herdecke University, Witten, Germany; 3 Department of Maxillofacial Surgery, RWTH Aachen University, Aachen, Germany

**Keywords:** Algorithm, Artificial intelligence, Craniofacial landmarks, Cone-beam computed tomography

## Abstract

**Objectives:**

Due to advancing digitalisation, it is of interest to develop standardised and reproducible fully automated analysis methods of cranial structures in order to reduce the workload in diagnosis and treatment planning and to generate objectifiable data. The aim of this study was to train and evaluate an algorithm based on deep learning methods for fully automated detection of craniofacial landmarks in cone-beam computed tomography (CBCT) in terms of accuracy, speed, and reproducibility.

**Materials and methods:**

A total of 931 CBCTs were used to train the algorithm. To test the algorithm, 35 landmarks were located manually by three experts and automatically by the algorithm in 114 CBCTs. The time and distance between the measured values and the ground truth previously determined by an orthodontist were analyzed. Intraindividual variations in manual localization of landmarks were determined using 50 CBCTs analyzed twice.

**Results:**

The results showed no statistically significant difference between the two measurement methods. Overall, with a mean error of 2.73 mm, the AI was 2.12% better and 95% faster than the experts. In the area of bilateral cranial structures, the AI was able to achieve better results than the experts on average.

**Conclusion:**

The achieved accuracy of automatic landmark detection was in a clinically acceptable range, is comparable in precision to manual landmark determination, and requires less time.

**Clinical relevance:**

Further enlargement of the database and continued development and optimization of the algorithm may lead to ubiquitous fully automated localization and analysis of CBCT datasets in future routine clinical practice.

## Introduction

Artificial intelligence (AI) deals with the understanding and imitation of human behavior. The aim is to cope with complex tasks and problems, as well as to automate object and word recognition [1].

The literature review shows that many disciplines in medicine are increasingly recognizing the benefits of AI for optimizing everyday work and are conducting more and more research on it [2–4].

Since orthodontists and oral and maxillofacial surgeons have a large clinical image database in the form of X-ray diagnostics, the use of AI in these specialties is of particularly great interest.

Through cephalometric measurements based on radiographs, oral and maxillofacial surgeons and orthodontists can analyze patient-specific jaw and skull geometry to diagnose craniofacial deformities, infer norm deviations, plan treatment, and simulate the outcome of potential surgery in advance [5–7].

Modern cone-beam computed tomography (CBCT) is a newer radiographic technique for imaging three-dimensional (3D) reconstructions and slice images.

Due to numerous limitations of two-dimensional (2D) radiographs, such as superimposition and distortion of anatomical structures and unequal magnification of bilateral structures, CBCT imaging is an increasingly common analytical tool in medicine and dentistry and, thus, a recent alternative for imaging cranial structures [8]. The diagnostic accuracy of CBCTs in dentistry has been widely investigated. The literature has shown that the detection rate of pathologies was significantly higher compared with conventional radiography and that cephalometric evaluation is accurate [9, 10].

A major disadvantage of cephalometric analysis when using CBCT images is that positioning anatomical landmarks in three slice planes is a more time-consuming procedure than conventional lateral cephalometric radiographs. In addition, the reproducibility of cephalometric analyses can vary from one physician to another and depends on medical expertise and the definition of landmarks [8, 11]. Cephalometric landmarks imaged by overlays in 2D projections are difficult to determine in 3D views.

Therefore, there have been increasing efforts to implement a fully automated landmarking system in routine clinical practice to assist clinicians by reducing the workload, which can potentially reduce errors and achieve more consistent results [12–21].

One of the central areas of AI is machine learning (ML). By repeatedly recognizing certain patterns, corresponding algorithms can be developed, and thus, decision-making can be made [22].

Deep learning is a particular type of ML that uses artificial neural networks and is a method of creating AI [2].

Artificial neural networks are highly interconnected networks of computer processors that are inspired by biological nervous systems [1]. The performance of convolutional neural networks (CNNs) depends on the number and quality of the available training datasets [23].

Whereas other fields of medicine have used a few thousand training datasets for the development of AI-based algorithms for automatic landmark positioning [24], recent research has investigated algorithms for automatic localization of cephalometric landmarks using significantly fewer image samples [12–21] .

The aim of the present study was to evaluate a novel algorithm regarding its applicability for the task of automatically detecting landmarks in large CBCT datasets and the accuracy in comparison to manually placed landmarks.

In addition to detecting cephalometric landmarks in CBCT datasets, we were interested in localizing the inferior alveolar nerve in the mandibular osteotomy line to minimize one of the main risks of mandibular bilateral sagittal split osteotomy (BSSO) and inferior alveolar nerve injury [25].

## Materials and methods

### Software and automatic landmark prediction

The newly developed software from the company Densilia® (Munich, Germany) that can be used for the automatic localization of craniofacial landmarks was implemented in the programming language Python. With this software, it was possible to visualize CBCT datasets and locate landmarks in three planes (coronal, sagittal and axial) manually and automatically.

The algorithm we studied is based on deep learning. In general, Densilia® uses a three-stage model with volumetric segments of different sizes. Each stage is build on the same architecture of the 3D U-Net algorithm. At each stage, an immense amount of information is collected about specific features of the craniofacial points in different layers and processed in connection points. At the first stage, the original image is represented by a volumetric segment of size 128×128×128 voxels and trained for 120 epochs. In this stage, rough positions of landmarks are first determined, which are then localized more precisely in the following two refinement stages. For this purpose, in step 2, the original image is scaled to 256×256×256 voxels and a section of 128×128×128 voxels is created around the respective rough position of the landmark from stage 1. In total, the algorithm is trained for 20 epochs at stage 2. By scaling down, the volume extracts are divided into smaller and smaller resolutions and the localization becomes more and more accurate. In stage 3, a volume of 128×128×128 voxels around the position of each landmark predicted in stage 2 is again extracted from the original CBCT, and new predictions are made for the landmarks. The inputs of each stage (and the outputs) are different and range from a general coarse to a specific accurate localization of the craniofacial landmarks. In this way, the maximum output of the CNN can be achieved. Better resolution and more precise localization of the landmarks than at stage 3 is no longer possible.

### Dataset

All CBCT datasets used in the current study were acquired between 2013 and 2020 in the Department of Oral and Maxillofacial Surgery. The CBCT datasets were collected retrospectively. Patient data were blinded; only age and sex were recorded. The CBCTs of 620 female (59.3%) and 425 male (40.7%) patients with an average age of 37.1 ± 19.7 years were analyzed. CBCT scans were acquired in DICOM (Digital Imaging and Communications in Medicine) format with the Galileos® Comfort Plus, Dentsply Sirona (Bensheim, Germany); the examinations were performed at 5 mA and 98 KV, with an effective radiation time of 14 s. The axial slice thicknesses was 0.287 mm and 0.250 mm, with isotropic voxels of 512×512×512 and 616×616×616. The inclusion criteria for the CBCT datasets were a large field of view (15.4 cm) and images with high morphologic variation and variability. The exclusion criteria were a poor resolution and CBCT images of patients with fractures, malformations, and visibly performed previous operations. In total, 156 CBCTs were excluded from a pool of 1201 CBCTs because of insufficient quality in terms of physical and diagnostic image quality. The datasets of 1045 CBCTs were split training, validation, and test set at a ratio of 8:1:1.

Of the analyzed CBCTs, 89% were used for training and validation and 11% for testing the algorithm.

Four independent orthodontists who were experts in their field participated in the study and helped train and validate the software with 931 (89%) different CBCT datasets. The collection and use of data were approved by the Institutional Ethics Committee of the Faculty of Medicine (EK 217/22).

### Landmarks

In the present study, 35 landmarks were selected from the hard tissue of the skull in each of the 1045 CBCT images to evaluate the manual precision of the experts and software for automatic landmark determination.

The landmarks varied in their difficulty of identification and represented midsagittal and bilateral anatomical features. Seven of the landmarks were located in the median plane, while 14 were distributed on the right and left sides of the maxilla, mandible, and midface.

The landmarks at different locations had, on average, very different localization errors. Therefore, the landmarks were divided into three categories:

1. Landmarks in the midsagittal plane of the skull (median landmarks)
2. Landmarks in the region of the bilateral cranial structures (bilateral landmarks)
3. Landmarks in the region of the osteotomy line of a bilateral sagittal mandibular split (osteotomy landmarks)

A detailed description of the landmark definitions is given in Table 1.

**Table 1:** Definition used for 3D landmarks

No.	Landmark	Abbreviation	Definition
1	Nasion	n	Most anterior located point of the sutura nasofrontalis.
2	Sella	s	Bone structure on the inner side of the Os sphenoidale, which divides the middle cranial fossa in the median plane.
3	Pogonion	p	Most anterior point of the bony chin in the median plane.
4	A- Point	a	Most dorsal point of the anterior maxilla.
5	B-Point	b	Most dorsal point of the anterior mandible.
6	Spina nasalis anterior	spa	Most anterior located point at the junction of the right and left maxillary bones.
7	Spina nasalis posterior	spp	Most dorsal located point at the junction of the right and left maxillary bones.
8	Condylus cranial right	c_cran_r	Most cranial point of the right caput mandibulae.
9	Condylus dorsal right	c_dors_r	Most dorsal point of the distal contour of the caput mandibulae right.
10	Ramus ascendens dorsal right	r_dors_r	Most anterior point of the processus angularis at the posterior margin of the ascending branch of the mandible on the right, directly below the caput mandible.
11	Tangent point P right	tg_p_r	Most posterior point of the processus angularis at the posterior margin of the ascending branch of the mandible on the right.
12	Tangent point A right	tg_a_r	Most caudal point of the processus angularis at the lower edge of the horizontal branch of the mandible on the right.
13	Menton right	m_r	Most anterior and inferior midpoint of the chin on the outline of the mandibular symphysis right.
14	Foramen mentale right	fmen_r	Hole located above the apex of the second premolar right. Entry point of the mental nerve from the mandibular canal right.
15	Mandibular foramen right	fman_r	Hole on the inner side of the ascending branch of the mandible right. Entry point of the nervus alveolaris inferior into the canalis mandibluae right.
16	Canalis mandibularis buccal right	cm_bucc_r	Most buccal point of the mandibular canal in the osteotomy line on the right.
17	Canalis mandibularis lingual right	cm_ling_r	Most lingual point of the mandibular canal in the osteotomy line on the right
18	Canalis mandibularis caudal right	cm_kcu_r	Most caudal point of the mandibular canal in the osteotomy line on the right.
19	Osteotomy point buccal right	ost_bucc_r	Most buccal point of the mandible in the osteotomy line on the right.
20	Osteotomy point lingual right	ost_ling_r	Most lingual point of the mandible in the osteotomy line on the right.
21	Osteotomy point caudal right	ost_cau_r	Most caudal point of the mandible in the osteotomy line on the right.
22	Condylus cranial left	c_cran_l	Most cranial point on the left caput mandibulae.
23	Condylus dorsal left	c_dors_l	Most dorsal point of the distal contour of the caput mandibulae left.
24	Ramus ascendens dorsal left	r_dors_l	Most anterior point of the processus angularis at the posterior margin of the ascending branch of the mandible on the left, directly below the caput mandible.
25	Tangent point P left	tg_p_l	Most posterior point of the processus angularis at the posterior margin of the ascending branch of the mandible on the left.
26	Tangent point A left	tg_a_l	Most caudal point of the processus angularis at the lower edge of the horizontal branch of the mandible on the left.
27	Menton left	m_l	Most anterior and inferior midpoint of the chin on the outline of the mandibular symphysis left.
28	Foramen mentale left	fmen_l	Hole located above the apex of the second premolar left. Entry point of the mental nerve from the mandibular canal left.
29	Mandibular foramen left	fman_l	Hole on the inner side of the ascending branch of the mandible left. Entry point of the nervus alveolaris inferior into the canalis mandibluae left.
30	Canalis mandibularis buccal left	cm_bucc_l	Most buccal point of the mandibular canal in the osteotomy line on the left.
31	Canalis mandibularis lingual left	cm_ling_l	Most lingual point of the mandibular canal in the osteotomy line on the left.
32	Canalis mandibularis caudal left	cm_cau_l	Most caudal point of the mandibular canal in the osteotomy line on the left.
33	Osteotomy point buccal left	ost_bucc_l	Most buccal point of the mandible in the osteotomy line on the left.
34	Osteotomy point lingual left	ost_ling_l	Most lingual point of the mandible in the osteotomy line on the left.
35	Osteotomy point caudal left	ost_cau_l	Most caudal point of the mandible in the osteotomy line on the left.

### Testing dataset and ground truth

To test the new software, 114 CBCTs (11%) of the 1045 CBCTs were randomly selected.

All 114 CBCTs were analyzed by three of the four experts in a random order.

The ground truth (GT) was labeled and based on the analysis of the fourth expert, who had more than 6 years of clinical and theoretical experience in cephalometry.

All four experts were experienced orthodontists and worked independently in their own private office or at a university.

To investigate the reproducibility of the manual landmark detection and, thus, the intraindividual variation of the individual experts, 50 CBCT datasets were unknowingly analyzed twice.

### Manual landmark identification

Before the viewing sessions, each expert received verbal and practical instructions and was trained in the use of five CBCT scans, which were not included in the present study.

CBCT datasets were displayed in a three-panel window containing sagittal, axial, and coronal multiplanar (MPR) views.

**Fig. 1:**
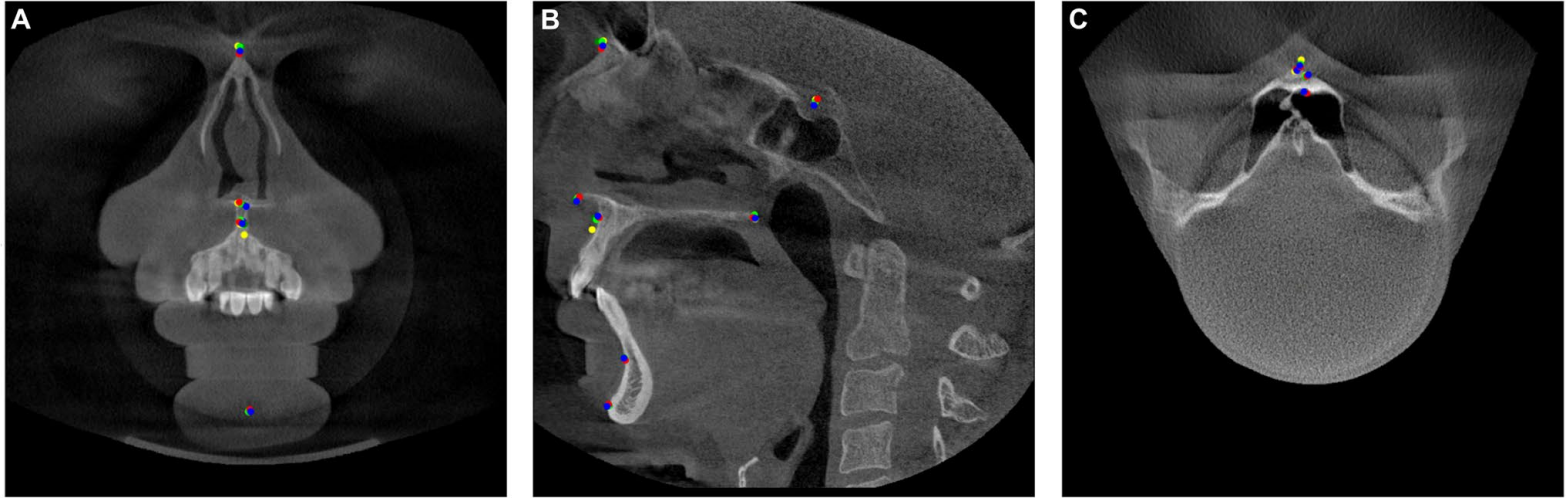
Sample of a CBCT image with median landmarks in coronal (**A**), sagittal (**B**), and axial (**C**) MPR view; red landmarks: GT; lime, green, yellow landmarks: experts 1, 2, 3; blue landmarks: AI

**Fig. 2:**
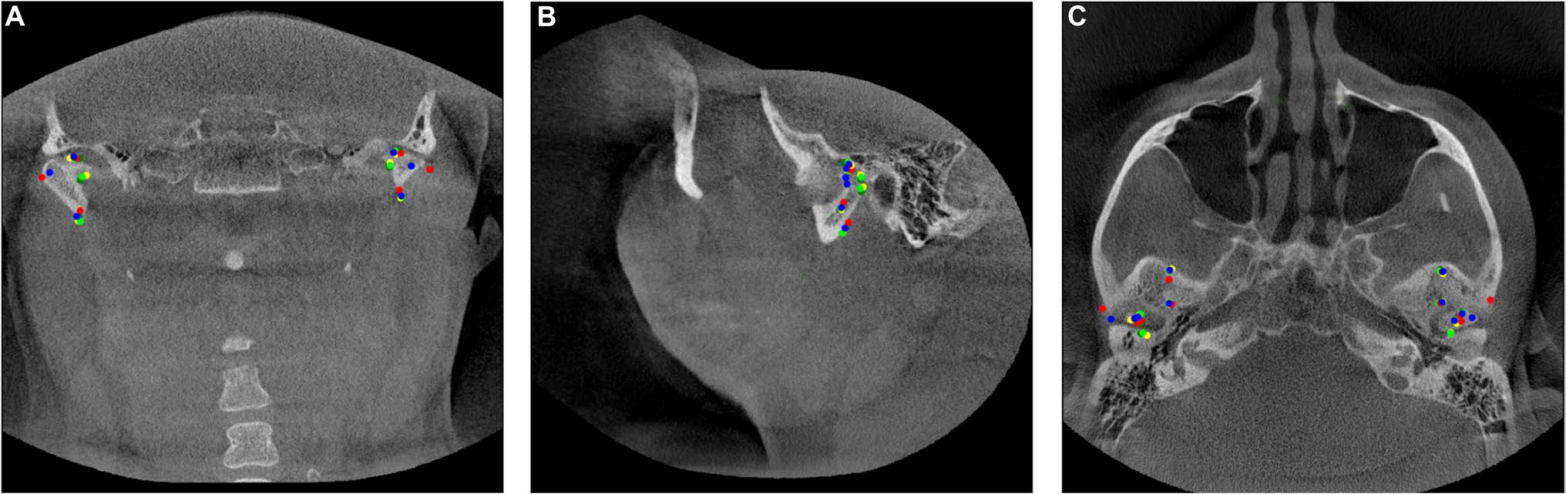
Sample of a CBCT image with paramedian landmarks in coronal (**A**), sagittal (**B**), and axial (**C**) MPR views; red landmarks: GT; lime, green, yellow landmarks: experts 1, 2, 3; blue landmarks: AI

**Fig. 3:**
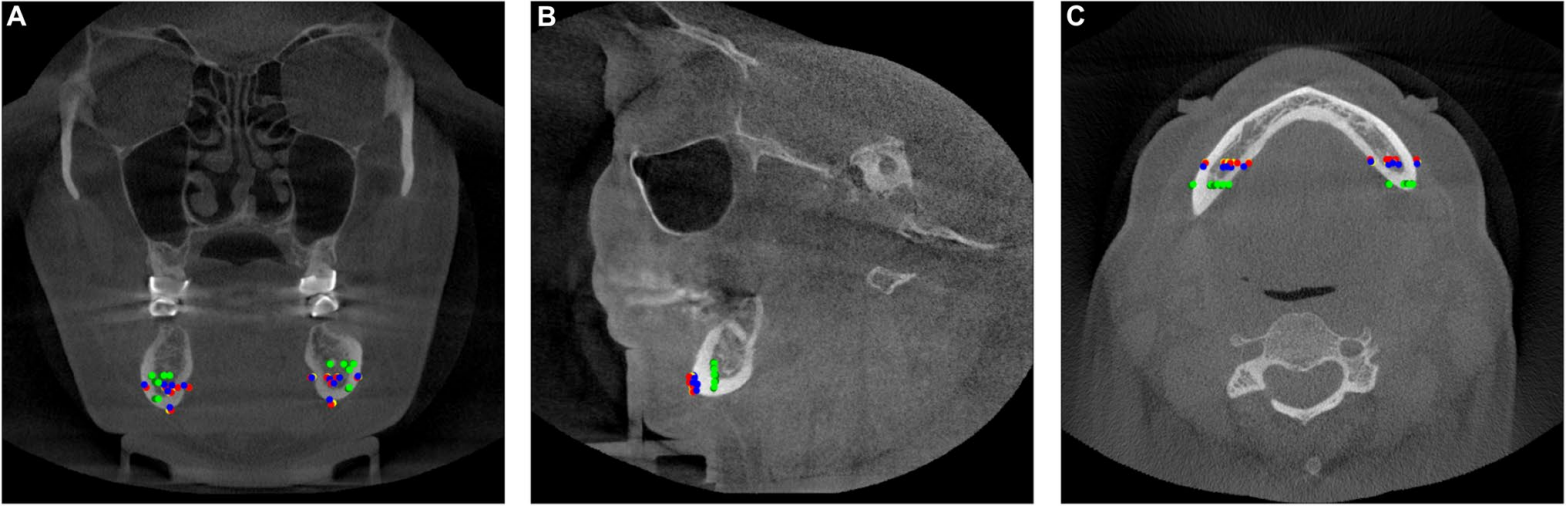
Sample of CBCT image with localized landmarks in the osteotomy line of the BSSO in coronal (**A**), sagittal (**B**), and axial (**C**) MPR views; red landmarks: GT; lime, green, yellow landmarks: experts 1, 2, 3; blue landmarks: AI

Independently and using the software, all four experts plotted landmarks using a graphical cursor at three different planes (sagittal, axial, coronal) of a CBCT dataset. Each landmark generated three coordinates in the *x*-, *y*-, and *z*-axes (Figures 1, 2, and 3).

Image enhancement features, such as zoom in/out and changes of brightness and contrast, were available for finding the landmarks more accurately.

### Statistical evaluation

Novel AI-based software for automatically locating landmarks in the CBCT datasets was tested for accuracy, speed, and reproducibility of results.

To evaluate the accuracy of the software in automatically detecting the landmarks, the GT was compared with the coordinates generated by the algorithm and with the mean values of three experts.

To compare CBCTs with different resolutions, pixels were converted to millimeters (mm).

Here, 0.25 mm was the pixel spacing in images with a resolution of 616×616×616, and 0.287 mm was the pixel spacing in images with a resolution of 512×512×512.

If multiple labels were available for an image, then only the first label was used because using the average of multiple labels would artificially reduce the errors and would not be representative of the real scenario.

**Fig. 4:**
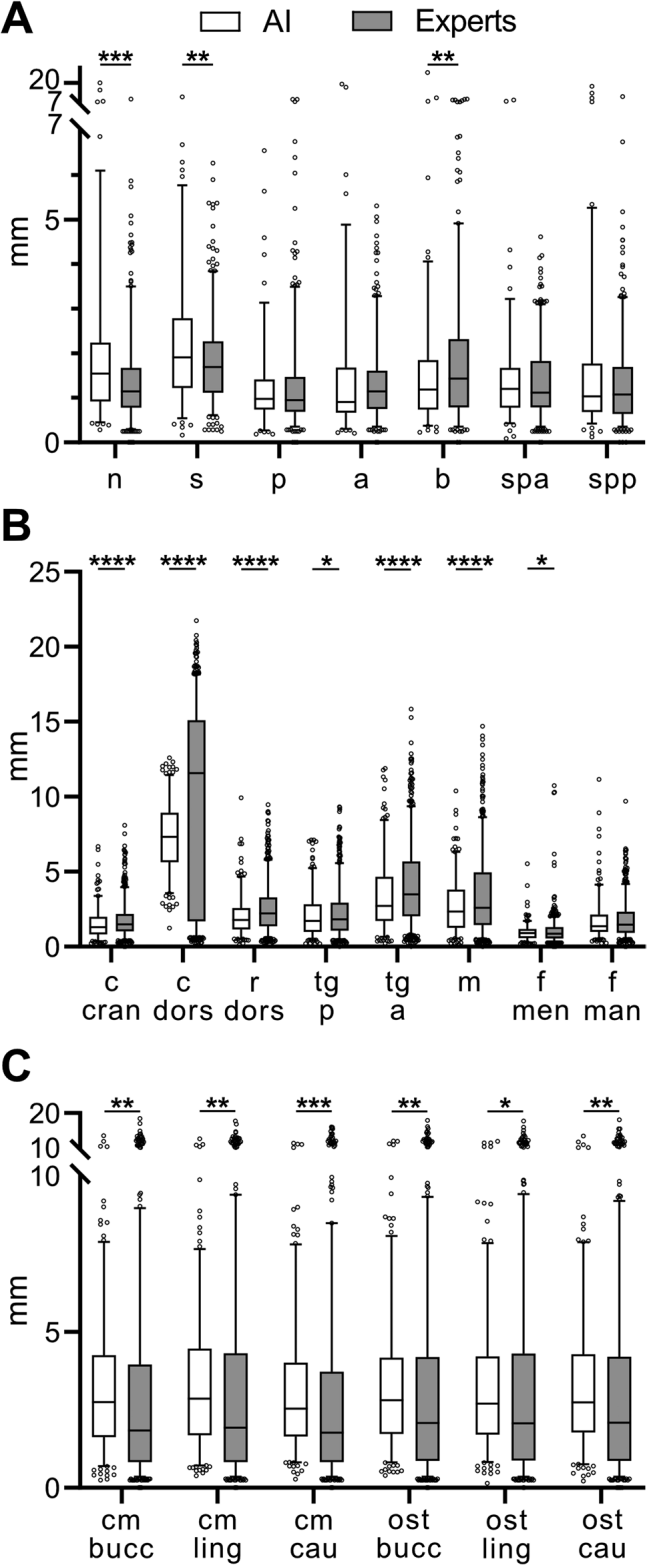
Boxplot for the Euclidean distance between the coordinates of each landmark in (**A**) midsagittal plane, (**B**) bilateral cranial structure, (**C**) osteotomy line with manual and automatic recognition of ground truth. The right and left values in (**B**) and (**C**) were combined. Box, 25^th^–75th percentile; line in box, median; whiskers, 5–95 percentile; statistically significant differences are marked with ****p < 0.0001; ***p < 0.001; **p < 0.01; *<0.05 ; *y*-axis: millimeter; *x*-axis: Abbreviations of landmarks explained in Table 1

The mean values were calculated for each landmark of manual and automatic detection, and the distance to GT was measured as an error in three spatial planes. The distance of the coordinates from the GT corresponded to the length of the shortest vector in space. The coordinates of each landmark were analyzed using SciPy (Python-based open-source software environment) analysis. Because the Shapiro-Wilk test did not indicate a normal distribution of the data, a comparison between human and machine accuracy at 114 different CBCTs was performed using the Wilcoxon matched-pairs signed-rank test. The Wilcoxon matched-pairs signed-rank test compared the AI with the mean of the three experts as paired samples of a non-normally distributed dataset. For Figure 4, the software GraphPad Prism 9.4.1 was used.

The combined error and normal combined error of the algorithm and three experts were determined in millimeters and percent.

We analyzed the significance of the results at different significance levels of 95%, 99%, 99.9%, and 99.99%. Hence, we considered a significant difference if the obtained *P*-values were smaller than 0.05, 0.01, 0.001, and 0.0001.

The time required by the experts to manually locate the landmarks was measured and compared with the speed of the algorithm.

A one-way ANOVA was used to determine the intraspecific variance of each expert and GT. The standard deviation (SD) indicated how high the true error of each physician could be at a confidence interval (CI) of 99%.

## Results

Table 2 shows both the total error in millimeters (mm) of the AI and landmarks manually identified by the experts for all landmarks combined, as well as the total error for the median, bilateral, and osteotomy landmarks.

**Table 2:** Results accuracy artificial intelligence versus experts

Results accuracy									
	AI	Experts		E1		E2		E3	
Combined error (mm)	2.73	2.79		2.79		2.79		2.78	
Combined error (%)	100%	+2.1%		+2.4%		+2.3%		+1.7%	
		*Z*=3145	*p*=0.71	*Z*=3244	*p*=0.92	*Z*=3146	*p*=0.71	*Z*=3227	*p*=0.88
Combined normal error (%)	100%	+ 0.8%		+0.6%		+0.8%		+1%	
		*Z*=3234	*p*=0.9	*Z*=3127	*p*=0.67	*Z*=3225	*p*=0.88	*Z*=3244	*p*=0.92
Median points (7 points) (mm)	1.75	1.5***		1.47***		1.51***		1.52**	
		*Z*=2103		*Z*=2035		*Z*=2111		*Z*=2273	
Osteotomy points (12 points) (mm)	3.27	2.93**		2.98**		2.93*		2.89**	
		*Z*=2340		*Z*=2331		*Z*=2398		*Z*=2332	
Other points (16 points) (mm)	2.75	3.24***		3.23***		3.25***		3.24***	
		*Z*=1702		*Z*=1763		*Z*=1760		*Z*=1711	

*AI*, artificial intelligence; *E1*, expert 1; *E2*, expert 2; *E3*, expert 3: combined error in mm and % as well as the combined normal error in % of the artificial intelligence and the three experts for all landmarks together as well as divided into three categories (median points, osteotomy points, other points). Statistically significant differences are marked with ***p < 0.001; **p < 0.01; *p < 0.05

The total error of all landmarks combined was 2.73 mm for the AI and 2.79 mm for the median experts.

The combined error in percentage shows that the experts made larger errors compared with the AI in 2.12% of the cases. The combined normal error gave a value of +0.8%.

Because the landmarks at different locations had, on average, very different localization errors, the landmarks were divided into three categories.

The mean error for the median points was 1.75 mm for the AI and 1.50 mm for the experts.

The median mean error of the osteotomy points was 3.27 mm for the AI and 2.93 mm for the experts. The mean error of the landmarks in the bilateral cranial structures combined was 2.75 mm for the AI and 3.24 mm for the experts.

The total error in the Wilcoxon rank test, which utilized all evaluated landmarks, shows that there was no significant difference at the corresponding significance levels between the experts and AI (*p* = 0.71). In detail, none of the three experts significantly differed from the AI (expert 1 (*p* = 0.92), expert 2 (*p* = 0.71), expert 3 (*p* = 0.88)).

Table 3 shows the errors of the three experts for each landmark in the median and AI, as well as the SD of both measurement methods. The most accurate landmark identified by the AI and experts was the mental foramen on the right side, with a median error of 0.88 mm (SD ± 0.42 mm) for the AI and total error of 0.94 mm (SD ± 0.53 mm) for the experts. The least accurate landmark identified by the AI and experts was the condyle dorsalis point on the left side, with a median error of 7.41 mm (SD ± 1.96 mm), which was similar to that of the experts who identified a total error of 9.15 mm (SD ± 6.82 mm).

**Table 3:** Overall performance of AI (artificial intelligence) and experts: mean absolute error (mm) of all landmarks with SD

Landmarks	Mean AI	SD AI (±)	Mean experts	SD experts (±)
n	2.13	2.53	1.44	1.07
s	2.29	1.63	1.83	1.00
p	1.26	0.99	1.29	1.15
a	1.88	3.42	1.34	0.88
b	1.56	1.40	1.86	1.58
spa	1.47	1.20	1.36	0.83
spp	1.70	2.30	1.35	1.08
c_cran_r	1.55	1.10	1.77	1.24
c_dors_r	7.23	2.56	8.87	6.66
r_dors_r	2.08	1.23	2.61	1.59
tg_p_r	2.02	1.40	2.14	1.48
tg_a_r	3.53	2.42	4.14	2.73
m_r	2.77	1.90	3.53	2.75
fmen_r	0.88	0.42	0.94	0.53
fman_r	1.54	0.98	1.62	1.05
cm_bucc_r	3.00	2.20	2.64	2.72
cm_ling_r	3.16	2.32	2.75	2.79
cm_cau_r	3.05	2.09	2.48	2.60
ost_bcck_r	3.14	2.27	2.74	2.71
ost_ling_r	3.12	2.34	2.75	2.70
ost_cau_r	3.14	2.19	2.75	2.69
c_cran_l	1.55	0.99	1.72	1.04
c_dors_l	7.41	1.96	9.15	6.82
r_dors_l	2.08	1.43	2.49	1.62
tg_p_l	2.21	1.57	2.36	1.74
tg_a_l	3.40	2.37	4.10	2.71
m_l	2.80	1.88	3.36	2.55
fmen_l	1.01	0.69	1.14	1.11
fman_l	1.97	1.67	1.95	1.28
cm_bucc_l	3.52	2.23	3.11	3.02
cm_ling_l	3.49	2.05	3.21	3.04
cm_cau_l	3.25	2.13	2.99	2.95
ost_bucc_l	3.49	2.10	3.26	3.06
ost_ling_l	3.40	2.00	3.26	3.01
ost_cau_l	3.46	2.19	3.24	2.97

Figure 4 illustrates the errors again in a boxplot diagram.

There were a total of seven boxplots, as shown in Figure 4A, for the medial landmarks, which, for the AI all lied within a similar error range between 1.26 mm (SD ± 0.99 mm) for the Pogonion landmark and 2.29 mm (SD ± 1.63 mm) for the Sella landmark. The error values of the averaged experts were also close, between 1.29 mm (SD ± 1.15 mm) at landmark Pogonion and 1.86 mm (SD ± 1.58 mm) at the landmark B-point. The bilateral cranial structures are shown in eight boxplots in Figure 4B. The error values of the bilateral structures of the right and left sides were combined into one value. The error values varied widely among both the AI and experts. Although the landmark condyle dorsalis showed an error value of up to 7.32 mm (SD ± 2.26 mm) in the AI, the experts reached an averaged error value of even 9.01 mm (SD ± 6.74 mm). The landmark with the smallest deviation was the mental foramen. The AI achieved an accuracy of up to 0.94 mm (SD ± 0.55 mm) and the experts an accuracy of 1.51 mm (SD ± 0.82 mm).

In contrast, the landmarks of the osteotomy line (Figure 4C) again showed more consistent results. Again, the values of the left and right sides were each combined as one value and, thus, have been presented in six boxplots. The error values for the AI ranged from 3.15 mm (SD ± 2.11 mm) for the canalis mandibularis caudal landmark to 3.32 mm (SD ± 2.18 mm) for the canalis mandibularis lingual landmark. The experts achieved the best result with an error value of 2.73 mm (SD ±2.77 mm), also at the landmark canalis mandibularis caudal, and the worst result with an error value of 3.00 mm (SD ± 2.85 mm), here at the landmark osteotomy point lingual.

As can be seen in Table 4, the experts and GT had an intraindividual standard error of the mean (SEM) of 0.6 mm for expert 1, 0.57 mm for expert 2, 0.6 mm for expert 3, and 0.74 mm for GT, while the AI always delivered the same result. With a CI of 99%, we can assume that the mean error in millimeters was 1.96 mm for GT, 1.55 mm for expert 1, 1.47 mm for expert 2, and 1.97 mm for expert 3.

**Table 4:** Intravariability of artificial intelligence, ground truth, and experts

Dataset	SEM	CI 99%	CI 99.9%
AI	± 0 mm	0 mm	0 mm
GT	± 0.76 mm	1.96 mm	2.5 mm
E1	± 0.60 mm	1.55 mm	1.97 mm
E2	± 0.57 mm	1.47 mm	1.88 mm
E3	± 0.60 mm	1.55 mm	1.97 mm

*SEM*, standard error of the mean; *CI 99%*, confidence interval 99%; and CI 99.9%, confidence interval 99.9% of AI (artificial intelligence), GT (ground truth), and three experts (E1, E2, E3)

Using the CI, we calculated the percentage of landmarks of each CBCT image, which were better detected by the AI than by the three experts.

The AI was more accurate in localizing landmarks than manual localization by the three experts in 28.5% of the cases, less accurate in 23.1% of the cases, and equal in 48.4% of the cases.

Expert 1 took a median of time (*t*) = 4.8 min, expert 2 *t* = 4.3 min, and expert 3 *t* = 4.0 min to evaluate and manually locate the 35 landmarks in each of the three spatial planes on the MPR view of a CBCT image, while the AI took 15 s to complete a full analysis.

## Discussion

Despite the significant technical progress of CBCT devices in recent years and the ever-increasing amount of image material, fully automated analysis of CBCT datasets is still in its early stages.

Most of the work described in the literature on the development of algorithms for automatic localization of landmarks in the head region has been based on 3D image registration [15, 17], knowledge-based [16, 19], learning-based [12, 14, 21], or hybrid learning- and knowledge-based approaches [18, 20]. The performance of an algorithm based on knowledge-based or registration-based methods can be questioned in the case of severely deformed skulls. Although the learning-based image registration method is sensitive to anatomical variations, the knowledge-based method has limitations in detecting the landmarks on curved structures. Therefore, in the present study, an innovative learning-based algorithm for the fully automatic detection of craniofacial landmarks in CBCT scans was investigated, presented, and evaluated in terms of accuracy, reproducibility, and speed.

The outcome of the learning-based approach correlated with the number, accuracy, and variability of the CBCT images included in the training set. Although we used 931 different CBCTs to train and validate the algorithm, comparable studies did not specify the training dataset [14–16, 18, 21]. Furthermore, studies on the automatic localization of landmarks in CBCT datasets have often been limited in their representativeness and accuracy because of the representation of the CBCT images, a small test dataset, and a selective choice of landmarks.

Unlike other studies [17], we chose to use a multiplanar view to display CBCT images because research by de Oliveira et al. showed that using constructed three-dimensional images from CBCT datasets alone can lead to errors in landmark location, which appear to be minimized by using multiplanar images [10].

With the exception of a recent study by Ghowsi et al. [21], which investigated an algorithm using 53 different landmarks in 100 CBCT scans, the maximum test dataset of comparable studies was 1–30 CBCT scans and 9–21 landmarks to be identified [12–20], some of which were localized only on the mandible [12] or midsagittal plane of the skull [14] and defined differently. In addition, some studies used the same datasets for training and testing, so their results are not necessarily comparable to our study [12, 13, 17]. The overall mean error of all automatically determined landmarks was 2.79 mm, which is well below the clinically acceptable error of up to 4 mm reported in previous publications [26]. It should be noted that this value was based on automatic detection of landmarks in 2D lateral radiographs and that the limit of acceptable error for 3D radiographs has not yet been evaluated [27–30].

Comparing the error of our method to the mean errors of the state of the art [12–21], which have ranged from 1.88 mm (SD ± 1.10 mm) in publications by Neelapu et al. [19] to 3.4 mm in Shadidi et al. [15], we have found that the accuracy of the landmarks all fell within a similar error range.

Because we found in our results that landmarks at different locations of the skull, on average, had very different error values, we not only examined the overall accuracy and the individual accuracy of each landmark, but we also divided them into three categories (median landmarks, bilateral landmarks, and osteotomy landmarks).

On average, the error value for median landmarks was 1.5 mm smaller than the error of the landmarks in bilateral cranial structures, which may be attributed to landmarks more difficult to define and localize. The median landmarks achieved a localization accuracy of 1.75 mm on average, which can be considered clinically correct according to the literature [26].

The literature has mainly described studies dealing with cephalometric landmarks.

We also investigated landmarks in the osteotomy line of a bilateral sagittal split of the mandible based on Obwegeser Dal-Pont, modified from Hunsuck Epker [31].

To reduce the risk of intraoperative iatrogenic damage to the inferior alveolar nerve in BSSO, it is critical for the surgeon to know the location of the nerve in the osteotomy line in the planning phase of dysgnathia surgery. With our study, we have pioneered a method for this. Although AI performed better in detecting landmarks in the midsagittal plane of the skull and bilateral cranial structures compared with experts, it showed a worse result in detecting landmarks in the osteotomy line of a bilateral sagittal mandibular split.

In general, the experts’ errors depended on individual experience, perceptual abilities, and differences in the effort required to localize landmarks [8, 32]. It is these three parameters that play an even greater role in the localization of landmarks on a defined but imaginary line, as they are considered very difficult to evaluate.

Due to the difficulty of localization, osteotomy landmarks have a high standard deviation. When the AI is trained with high variance data, there may be inaccuracies in the AI’s identification of these points.

In automatic generation, the landmark with the lowest error was the mental foramen, with an overall average error of 0.87 mm (SD ± 0.42 mm). This landmark can be accurately defined anatomically, allowing for less individual interpretation. The landmarks with the largest errors were mainly located in the mandibular region. One explanation for this could be that the mandible was statistically one of the most morphologically variable cranial bones [31]. The landmark condyle dorsalis shows that experts do not fully agree on the localization of landmarks. The SD was very high for the experts at ± 6.74 mm, while the AI gave a more consistent result, with a SD of only ± 2.26 mm, without any significance. As noted by Gupta et al., the identification of the mandibular condyle can be difficult because of its unique contour and shape, and landmarks located on a long, wide anatomical surface or on a protrusion of a curvature are subjective and difficult to find [16].

A single analysis of the 114 CBCT images by each of the three experts allowed for only a relative evaluation of each expert’s performance and a comparison with the AI. If the analysis of a CBCT dataset by the same expert was repeated several times, the variables would appear in the analysis. Although some studies in the literature only considered the error score obtained, our study has also examined the individual errors of each expert. By calculating the intraindividual variance of each expert, not only was the error value of the expert, but also the error of the AI, put into perspective. SEM and CI indicated how high the “true error” of the expert’s performance could be based on the available data. On average, the experts showed an intraindividual variance with a SEM of up to 0.6 mm, while the AI allowed an invariant analysis of the structures.

One of the major difficulties in superimposing CBCT images to monitor progress in treatment was to reliably reproduce the reference planes [33]. By using an automatic analysis based on objectifiable criteria, the reference planes can be consistently recognized. This may lead to a more reliable treatment prognosis and better control of the progress of treatment.

The manual localization of landmarks requires a lot of time, leading to clinicians’ visual tiredness. The automatic algorithm found the 35 landmarks on average were 95% faster than the experts, hence providing a reduction in clinical workload.

Another clinically relevant topic is that many clinicians are overloaded with the amount of additional information provided by 3D diagnostics compared with 2D diagnostics, presenting them with major challenges in analysis and treatment planning. Automating the analysis of 3D diagnostics opens up a wide field of diagnostics and makes them more accessible to clinicians, favoring the shift from 2D to 3D imaging in everyday clinical practice.

Although the algorithm evaluated in the current study has provided a valuable method for fully automated localization of craniofacial landmarks on CBCT images, the study has potential for discussion regarding methodology. The GT on which the algorithm was tested was based on the knowledge of a single orthodontist derived from human anatomy. At this stage, there is no technology that can accurately determine the position of the landmark. Until then, we must rely on the human eye of an expert as the gold standard. One possibility would be to retest the algorithm using the averaged values of several experts. However, this would require an enormous amount of organization and time because several experts would have to label the entire dataset in a consilium.

Previous studies have found that the accuracy of an AI-based algorithm correlates with the size of the dataset. The larger the amount of data, the more accurate the algorithm [32].

Based on our experience from previous studies, the amount of data that we used in the present study was just above the threshold to allow for sufficiently effective AI training. This finding was more pronounced for landmarks at the edge of the image, as the AI had more difficulties to detect landmarks in these areas. However, there is no well-defined threshold for sufficient accuracy, as this does not only depend on the amount of data, but also the quality of the ground truth data as well as the required accuracy for the intended purpose. The recruitment of large-volume CBCT datasets was not straightforward because of the limited use of CBCT scans in routine clinical practice. Standard routine diagnostics still include 2D lateral radiographs because these are associated with a lower radiation dose to the patient, which is especially a major concern in pediatric patients. CBCT is recommended as a complementary diagnostic method for certain indications that facilitate treatment planning based on 3D imaging [8]. Consideration should be given to adding datasets from different international centers to both expand the training, validation, and testing set and test the algorithm on skull morphologies of different ethnicities.

## Conclusion

Except for a few landmarks, the presented learning-based algorithm generated clinically acceptable mean error distances and, thus, can lead to a validation and, if necessary, correction of the individually made assessment based on objective criteria. The system-immanent comparison with the existing database increased reproducibility and, thus, can possibly lead to more reliable and faster diagnoses in the future, though further development is necessary.

We believe that the investigated algorithm has the potential to advance the fully automated analysis of craniofacial landmarks in CBCT images.

## Data Availability

The data underlying this article will be shared on reasonable request to the corresponding author.
